# Divergence between neural and retinal lineage specification during human brain development by signal transduction

**DOI:** 10.1016/j.jare.2025.10.034

**Published:** 2025-10-22

**Authors:** Ki Hong Nam, Sang Ah Yi, Lin-Fan Xiao, Jae Sung Noh, Min Gyu Lee, Jae Kyun Jeong, Hyewon Jung, Ning-Yi Shao, Jeung-Whan Han, Jaecheol Lee

**Affiliations:** a School of Pharmacy, Sungkyunkwan University, Suwon 16419, Republic of Korea; b Cell Biology Program, Memorial Sloan Kettering Cancer Center, New York, NY 10065, USA; c Department of Biopharmaceutical Convergence, Sungkyunkwan University, Suwon 16419, Republic of Korea; d Department of Biomedical Sciences, Faculty of Health Sciences, University of Macau, Macau Special Administrative Region (SAR), People's Republic of China; e Epigenome Dynamics Control Research Center (EDCRC), Sungkyunkwan University, Suwon 16419, Republic of Korea; f MoE Frontiers Science Center for Precision Oncology, University of Macau, Taipa, Macau SAR, China; g Biomedical Institute for Convergence at SKKU (BICS), Sungkyunkwan University, Suwon 16419, Republic of Korea; h Department of Biohealth Regulatory Science, Sungkyunkwan University, Suwon 16419, Republic of Korea

**Keywords:** S6K1, Human brain development, Brain organoids, Neuronal lineage specification, Retinal cell emergence

## Abstract

**Introduction:**

The physiological functions of p70 S6 kinase 1 (S6K1) have been extensively studied in S6K1-deficient mice. However, there is limited evidence demonstrating the influence of S6K1 deletion on human brain development.

**Objectives:**

In this study, we identify the role of S6K1 in human brain development utilizing genetically engineered human embryonic stem cell-derived brain organoids.

**Methods:**

Dorsal forebrain organoids generated from S6K1-depleted human embryonic stem cells (hESCs) were analyzed through single-cell RNA sequencing at early (5 weeks) and late (14 weeks) stages. In addition, the brain organoids derived from co-cultured S6K1-deleted and wild-type hESCs were subjected to ATAC-sequencing.

**Results:**

Genetic deletion of S6K1 significantly decreases the size of the dorsal forebrain organoids in the early stages. Single-cell RNA sequencing analysis shows an abnormal emergence of retinal cell lineages in S6K1-deleted brain organoids, which diverges from cortical neurons in the early stage, eventually leading to a decrease in the proportion of mature cortical neurons. The chromatin accessibility analysis of co-cultured brain organoids shows that retinal specification in S6K1 knockout organoids was due to non-cell-autonomous function, whereas incomplete maturation of neurons results from cell-autonomous function.

**Conclusion:**

Depletion of S6K1 signaling in the early stage of human brain development drives the formation of retinal cells distinct from cortical neurons. Our findings demonstrate that S6K1 signaling fine-tunes neuronal and retinal lineage specification during brain development.

## Highlights

• Deletion of S6K1 during human brain organoid development induces microcephaly.
• S6K1 deletion induces dual lineage specification into retinal and cortical neurons.
• The mature neurons were deprived in late stage of S6K1-depeleted brain organoids.
• The effects of S6K1 stem from both cell autonomous and non-cell autonomous action.

## Graphical abstract

**Figure:**
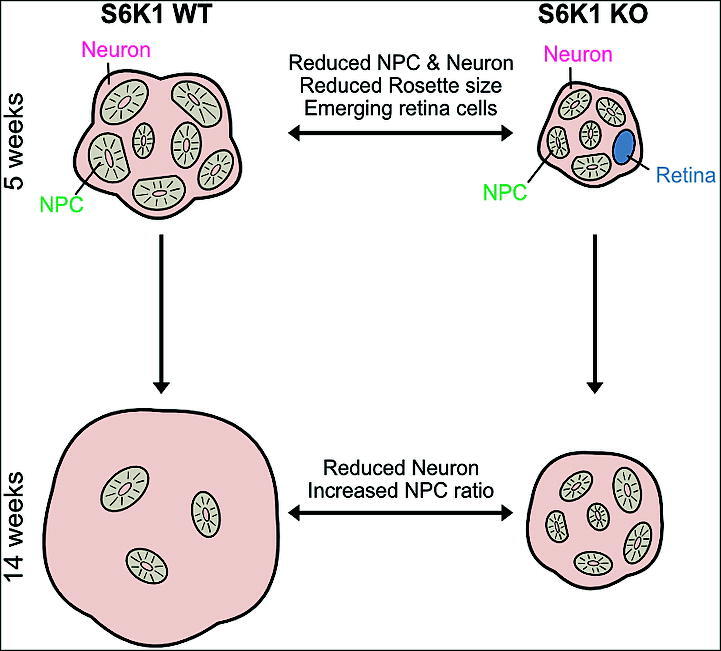

## Introduction

Multiple phases of brain development involve cell proliferation, cell lineage specification, migration, maturation, and axis patterning. The signaling pathways contributing to these processes, including the nodal, Wnt, and sonic hedgehog (SHH) signaling pathways, undergo activation or silencing at the correct time, space, and intensity during brain development. Dysregulation of these signaling events causes numerous neurodevelopmental malformations, such as macrocephaly, microcephaly, intellectual disability, and autism spectrum disorders. Therefore, it is important to identify the critical controllers for each developmental stage to accurately understand the pathogenesis of neurodevelopmental diseases.

The mammalian target of rapamycin (mTOR) signaling pathway is regarded as a key regulator of several conserved cellular outcomes, such as cell growth, translation, autophagy, and lipid synthesis through the activation of its direct downstream molecule, p70 S6 kinase 1 (S6K1) [1]. Our previous study identified the critical role of S6K1 in mesenchymal cell fate determination, demonstrating an anti-obesity phenotype in S6K1-deficient mice [2]. In addition to adipose mass, the weight of several other organs, including the brain, kidney, and spleen, were significantly reduced in S6K1 knockout mice [3]. Although it has long been established that the activity of S6K1 contributes to the development of mouse embryos [4], the accurate function of S6K1 in the early developmental stage of humans has not been fully understood. Recent clinical studies have shown that patients with MTOR gain-of-function mutations exhibit neurodevelopmental symptoms characterized by macrocephaly, seizures, intellectual disability, and cortical dysplasia [5,6]. In contrast, mTOR deficiency in the mouse brain induces microcephaly and reduces the number of neural progenitors [7,8]. However, the brain-specific knockout of S6K1, a downstream substrate of mTOR, did not alter the size of the mouse brain [3]. Notably, a major difference between mouse and human brains is the absence of an outer radial glial (oRG) layer, in which the active mTOR/S6K1 pathway is enriched [9,10].

Brain organoids derived from human pluripotent stem cells (hPSCs) mimic the developmental processes and functional features of the human brain. As conventional brain organoid models lack other cell types such as astrocytes, microglia, and capillaries, and mainly resemble cortical development, brain organoid models have been used for disease modeling related to neurodegeneration and brain malformation. Genetic engineering of PTEN, RAB39b, or TSC1/2 mutations can generate macrocephalic brain organoids with excessive expansion of neural progenitors [[11], [12], [13]]. In addition, by producing organoids from genetically mutated hPSCs or patient-derived iPSCs, researchers have identified several mutations leading to microcephaly and revealed a pathogenetic mechanism contributing to the phenotype [14,15].

Here, we elucidated the role of S6K1 in human brain development by generating brain organoids from S6K1-knockout (S6K1^-/-^) hESCs. We showed that deletion of S6K1 results in microcephaly with smaller neural rosettes during the initial stage of brain organoid differentiation. Single-cell transcriptomic analysis revealed unusual retinal cell development in S6K1-deleted brain organoids, thereby reducing neural populations. Furthermore, we generated mixed brain organoids containing both WT and S6K1^-/-^ hESCs and isolated them after complete differentiation to compare the features of each portion grown in mixed conditions. ATAC sequencing analysis of each portion in mixed brain organoids showed no significant differences in retinal cell marker gene transcriptomes between the WT and S6K1^-/-^ portions, while chromatin accessibility of neural-specific genes was lower in the S6K1^-/-^ portion compared to the WT portion. Collectively, our study revealed that S6K1 is responsible for the lineage specification of human brain development by restraining retinal differentiation and accelerating neuronal maturation, which are non-cell-autonomous and cell-autonomous actions, respectively.

## Material and Methods

### Human embryonic stem cell culture

The H7 human embryonic stem cell line was obtained from the WiCell Research Institute (WA07). H7 cells were grown under feeder-free conditions on Matrigel (Corning, 354234)-coated plates with TeSR-E8 medium (Stem Cell Technologies, 05990). The medium was refreshed every day. Cells were maintained in a fully humidified atmosphere of 95 % air and 5 % CO2 at 37 °C.

### Generation of S6K1 knockout cell line

To generate S6K1 knockout H7 ESCs, we targeted the exons as in a previous study [16]. The guide RNAs targeting exon 9 (E9-For: TTAAACATAATTGGTTTGGGGG, E9-Rev: GCAGCTTTTAACTGTGCACCTA) and exon 11 (E11-For: AAAAGAAATGCTGCTTCTCGTC, E11-Rev: AGGAAGAAAAGTTGAGGGGACT) of S6K1 were designed using an online CRISPR design tool (https://chopchop.cbu.uib.no/) and cloned into the pSpCas9(BB)-2A-GFP (PX458) (Addgene, #48138). Then, 1 μg of cloned plasmids was transfected into H7 ESCs using Lipofectamine 3000 reagent (Thermo Fisher Scientific, L3000008). After two days, GPF-expressing transfected cells were sorted using a BD FACS Aria cell sorter. The sorted cells were seeded on 6 well plates and expanded clonally. To confirm the genotype of each clone, genomic DNA was isolated using the DNeasy Blood & Tissue Kit (Qiagen, 69504), and PCR was conducted using PrimeSTAR GXL DNA Polymerase (TaKaRa, R050A).

### Brain organoid differentiation

Dorsal forebrain organoids were generated from H7 cells as previously reported [17]. H7 cells grown to 80 % confluency were dissociated using Accutase (Gibco, A11105-01) and reaggregated in ultra-low-cell adhesion 96 well V-bottom plates (S-bio, MS-9096VZ) (10,000 cells per well) with 100 mL per well of cortical differentiation medium (CDM I) (Glasgow-MEM (Gibco, 11710035) containing 20 % KSR (Gibco, 10828–028), 0.1 mM MEM-NEAA (Gibco, 11140–050), 1 mM sodium pyruvate (Gibco, 11360–070), 0.1 mM 2-mercaptoethanol (Gibco, 21985–023), and penicillin/streptomycin (Gibco, 15140–122)) supplemented with 20 μM Y-27632 (Tocris, Y0503) (day 0–6), 3 μM IWR-1 (Tocris, 3532) (day 0–18), and 5 μM SB431542 (Tocris, 1614) (day 0–18). The medium was changed every 3 days until day 18. From day 18 to day 34, cell aggregates were moved to 60 mm ultra-low-attachment culture dishes on the orbital shaker (70 rpm) in CDM II (DMEM/F12 containing 2 mM Glutamax (Gibco, 35050–061), 1 % N2 (Gibco, 17502–048), 1 % CD lipid concentrate (Gibco, 11905–031), 0.25 μg/ml fungizone (Gibco, 15290–018), and penicillin/streptomycin). From day 35 to day 69, organoids were incubated with CDM III (CDM II with 10 % FBS (Welgene, S001-01), 5 μg/ml Heparin (Sigma-Aldrich, H3149), and 1 % Matrigel (Corning, 354234)). From day 70, organoids were incubated CDM IV (CDM III with B27 (Gibco, 17504044) and 2 % Matrigel). CDM II, CDM III, and CDM IV were changed every 2 days. The size and shape of organoids were observed and visualized with Cytation 5 Cell Imaging Multi-Mode Reader (BioTek).

### Monolayer differentiation into cortical neuron

Directed differentiation of H7 cells into cortical neurons was performed as previously reported [17]. H7 cells were dissociated with 0.5 mM EDTA and plated at a high density. On the next day, the medium was changed to neural induction medium every day until day 8. At day 8, the neuroepithelial sheet was carefully lifted off with Dispase (Stem Cell Technologies, 07923), broken up gently into small aggregates of 300–500 cells, and plated on poly-L-ornithine (Sigma-Aldrich, P4957) and laminin (Sigma-Aldrich, L2020)-coated plates in neural induction medium. At day 9, the media was changed to neuronal maintenance medium supplemented with 20 ng/mL Fibroblast growth factor 2 (FGF2) (PeproTech, 100-18B). The medium was changed every other day, and FGF2 was removed from day 13. When neuronal rosettes appeared, the cells were split with Dispase at a 1:2 ratio. Neurons were expanded when they reached 90 % confluency until day 30. The neuronal maintenance medium consisted of 500 mL Neurobasal medium (Gibco, 21103049), 500 mL DMEM:F12 + Glutamax (Gibco, 10565018), 0.25 mL Insulin (10 mg/ml, Sigma-Aldrich, 19278), 1 mL 2-mercaptoethanol (50 mM), 5 mL MEM-NEAA (100 X), 5 mL sodium pyruvate (100 mM), 2.5 mL penicillin/streptomycin (10000 U/μl), 5 mL N2, 10 mL B27, and 5 mL Glutamax (100 X). For preparation of neural induction medium, 1 μM Dorsomorphin (Tocris, 3093) and 10 μM SB431542 were added to the neuronal maintenance medium.

### Antibodies

The following antibodies were used in this study and obtained from the indicated sources: Rabbit polyclonal anti-phospho-p70 S6 Kinase (Thr389) (Cell Signaling Technologies, 9205), Rabbit polyclonal anti-p70 S6 Kinase (Cell Signaling Technologies, 9202, for immunoblotting), Mouse monoclonal anti-p70 S6 Kinase (Santa Cruz Biotechnology, sc-8418, for immunocytochemistry), Rabbit monoclonal anti-phospho-S6 Ribosomal Protein (Ser235/236) (Cell Signaling Technology, 4858), Rabbit polyclonal anti-histone H3 (Abcam, ab1791), Rabbit polyclonal anti-HOPX (Proteintech, 11419–1-AP), Rabbit polyclonal anti-SOX2 (Abcam, ab59776), Rabbit polyclonal anti-MAP2 (EMD Millipore, ab2290), Mouse monoclonal anti-Brn-3/BRN3/POU4F (Santa Cruz Biotechnology, sc-390780), Mouse monoclonal anti-PAX6 (Abcam, ab78545), Mouse monoclonal anti-Nestin [2C1.3A11] (Abcam, ab18102), Mouse monoclonal anti-beta III Tubulin (Abcam, ab78078), Mouse monoclonal anti-EphA2 (C-3) (Santa Cruz Biotechnology, sc-398832), Rabbit polyclonal anti-OCT4 (Abcam, ab19857), Mouse monoclonal anti-acetylated alpha tubulin (Santa cruz Biotechnology, sc-23950), Goat anti-Rabbit IgG (H + L) secondary antibody, TRITC (Thermo Fisher Scientific, A16101), Goat anti-Mouse IgG (H + L) secondary antibody, Alexa Fluor 488 (Thermo Fisher Scientific, A11001), Goat anti-Mouse IgG (H + L) secondary antibody, Alexa Fluor 594 (Thermo Fisher Scientific, A11032), Goat anti-Rabbit IgG (H + L) secondary antibody, HRP (Thermo Fisher Scientific, 31460), Goat anti-Mouse IgG (H + L) secondary antibody, HRP (Thermo Fisher Scientific, 31430), DAPI (Santa Cruz Biotechnology, sc-3598), Phalloidin-iFluor 488 reagent (Abcam, ab176753).

### Immunoblotting

Protein lysates were extracted using the PRO-PREP^TM^ Protein Extraction Solution (Intron Biotechnology, 17081). Each protein sample was subjected to sodium dodecyl sulfate–polyacrylamide gel electrophoresis (PAGE) for size separation. The separated proteins were transferred to polyvinylidene difluoride (PVDF) membranes using a semi-dry transfer system (Bio-Rad). Membranes were incubated with primary antibodies overnight at 4 °C, followed by incubation with horseradish peroxidase (HRP)-conjugated secondary antibodies for 1 h at room temperature. HRP signals were detected using chemiluminescence reagents (Abclon, ABC-3001) on AGFA medical X-ray film blue (CP-BU).

### Immunohistochemistry

Immunohistochemistry of the sectioned organoids was performed as previously described [17]. For cryosectioning, organoids were fixed with 4 % formaldehyde in PBS for 20 min, gently washed twice with PBS, and equilibrated with 30 % sucrose in PBS at 4 °C until the organoids floated to the surface. The equilibrated organoids were embedded in Tissue-Tek O.C.T compound (Sakura Finetek, 4583) and cryosectioned to a 16 μm thickness. Each section was washed with 0.1 % Triton X-100 in PBS and incubated with blocking solution (5 % donkey serum and 0.1 % Triton X-100 in PBS) at room temperature for 1 h. Next, the sections were incubated with primary antibodies (diluted with 2.5 % donkey serum and 0.1 % Triton X-100 in PBS to 1:500) overnight at 4 °C followed by incubation with secondary antibodies (diluted 1:600) at room temperature for 1 h. After three washes (10 min each) with PBS, the sections were incubated with diluted DAPI (1:1000) at room temperature for 20 min. Fluorescence signals were detected using a Cytation 5 Cell Imaging Multi-Mode Reader (BioTek) or a TCS SP8 HyVolution confocal microscope (Leica).

### Immunocytochemistry

Immunocytochemistry of embryonic stem cells and monolayer cortical neurons was performed, as previously described [17]. The cortical neurons were fixed with 4 % paraformaldehyde in PBS for 15 min, followed by permeabilization with 0.1 % Triton X-100 in PBS for 15 min. The cells were blocked with 1 % bovine serum albumin (BSA) in PBS at room temperature for 1 h and incubated with primary antibodies (diluted with 1 % BSA in PBS to 1:500) overnight at 4 °C, followed by incubation with secondary antibodies (diluted with 1 % BSA in PBS to 1:600) at room temperature for 1 h. EdU + cells were detected using the Click-iT^TM^ EdU Cell Proliferation Kit for Imaging, Alexa Fluor^TM^ 488 dye (Thermo Fisher Scientific, C10337) according to the manufacturer’s instructions. Cell nuclei were stained with DAPI (1:1000) at room temperature for 15 min. Immunofluorescence signals were detected using a TCS SP8 HyVolution confocal microscope (Leica, Wetzlar, Germany).

### Bulk RNA sequencing of undifferentiated embryonic stem cells

Total RNA for bulk RNA sequencing was extracted from undifferentiated wild-type and S6K1-depleted embryonic stem cells using the miRNeasy kit (Qiagen, 217084). Library construction was conducted using the TruSeq Stranded mRNA Sample Preparation Kit (Illumina), according to the manufacturer’s instructions. High-throughput sequencing was conducted as paired-end 150 sequencing, using the MGISEQ-2000 platform (LAS, Seoul, Korea). The paired-end reads were trimmed and filtered with default parameters via trim_galore v0.6.5 software and then aligned to the human genome GRCh38 (hg38) using Bowtie2 v2.4.5 [18,19]. Aligned reads were converted into the bam format and sorted using Sambamba v0.8.2, followed by feature counts for quantification. The differentially expressed genes were detected using the DESeq2 v1.37.4 R package with the threshold adjusted p-value < 0.001, and the functional enrichment test was conducted using the GeneAnswers v1.16.0 R package (p-value < 0.05) [[20], [21], [22], [23]].

### Dissociation of organoids and single-cell RNA-sequencing

For single-cell RNA sequencing, 5 weeks or 14 weeks-old organoids were dissociated using a Papain dissociation kit (Worthington Biochemical Corporation, #LK003150), according to the manufacturer’s instructions. Briefly, chopped organoids were placed in 1 mL of papain solution and incubated at 37 °C for 30 min with inversion every 10 min. Next, the samples were gently triturated using a pipette and centrifuged at 300 × g for 5 min. After discarding the supernatant, the cells were resuspended in DNase diluted albumin-ovomucoid inhibitor solution (3.15 mL) and centrifuged at 70 × g for 6 min. The supernatant was discarded, and the pellet was resuspended in DPBS for scRNA sequencing.

Single cell droplets were barcoded using the Chromium Next GEM Single Cell 3′ Kit v3.1 (10x Genomics, cat# 1000121) by subjecting the cells to a chromium single cell controller (PN-120263;120212). A total of 10,000 cells was collected, and cDNA with barcodes was synthesized through reverse transcription in the droplet. The barcoded libraries were sequenced according to the manufacturer's instructions using an Illumina NovaSeq6000 (2 × 150 bp).

### Single-cell RNA-sequencing data analysis

Cells produced by the Chromium Next GEM Single Cell 3ʹ v3.1 kit from 10X Genomics were aligned to the human GRCh38 transcriptome using the 10x Genomics Cell Ranger 3.1.0, and all procedures were guided by 10x Genomics protocols with default parameters [24]. The analysis workflow, including quality control, normalization, feature selection, integration, scaling, dimensional reduction, unsupervised clustering, and differential expression, was performed using Seurat v 4.0.6 on RStudio, and pseudotime trajectory analysis was conducted using the Monocle3 R package [[25], [26], [27], [28], [29], [30], [31]].

Samples were filtered using certain thresholds (500 < nFeature_RNA < 7,000 and 500 < nCount_RNA, 5 %<ribosomal reads, and mitochondrial reads < 20 %). Normalization and identification of 3000 variable features for each dataset were performed using the “vst” method, followed by different integration for different analyses.

For specific time point analysis, samples from the same time point were integrated. Uniform manifold approximation and projection (UMAP) dimensional reduction was then computed using the selected significant principal components (PCs) (20 for each time point) after scaling (the percentage of mitochondria and ribosomes were treated as variables to regress out). Clustering of the cells was performed using FindNeighbors and FindClusters to choose the best resolution (0.2 for the 5th week and 0.3 for the 14th week). A differentially expressed gene test was conducted using the FindAllMarkers function with the default parameters, except that the parameter “only.pos” was true.

For pseudotime trajectory analysis, samples were integrated across biological conditions and time points. Scaling, dimensional reduction, and differential gene expression were the same as those described above, except that the resolution of unsupervised clustering was 0.3. The cells clustered by UMAP were separated via biological conditions and then were used to infer the cell differentiation trajectories by as.cell_data_set, cluster_cells with these parameters (reduction_method= “UMAP,” k = 20) and learn_graph command imported from the Monocle3 R package.

### Integrated analysis with public single-cell RNA-sequencing data

The organoid from the 5th week and public retina and RPE samples were individually filtered using specific thresholds. Subsequently, normalization and identification of 3000 variable features were performed for each dataset using the 'vst' method. To reduce dimensionality, UMAP was also computed using 40 PCs after scaling. The percentage of mitochondria and ribosomes were treated as variables for regression. Clustering of the cells was carried out using FindNeighbors and FindClusters, with an unsupervised clustering resolution set at 0.3. Differential gene expression analysis was conducted using the FindAllMarkers function, employing default parameters, except for setting the parameter 'only.pos' to true.

### Bulk quantitative real-time PCR (RT-qPCR)

For RNA extraction, organoids were lysed with the easy-BLUETM Total RNA Extraction Kit (Intron Biotechnology, 17061) on ice. RNA (1 μg) was reverse-transcribed into cDNA using a Maxime^TM^ RT PreMix (Random Primer) (Intron Biotechnology, 25082). Quantitative real-time PCR was performed using KAPA^TM^ SYBR FAST qPCR (KAPABIOSYSTEMS, KK4604) with a CFX96^TM^ or Chromo4^TM^ real-time PCR detector (Bio-Rad). Relative mRNA levels were normalized to 18S rRNA values for each reaction. The qPCR primer sequences used in this study are listed in Supplementary Table S1.

### ATAC-sequencing of fluorescence-labeled organoids

S6K1 knockout H7 cells were labeled with eGFP through infection with eGFP-expressing AAV2 viral particles (Gene Copoeia, AA001), followed by fluorescence-activated cell sorting (FACS). Dorsal forebrain organoids were generated using a 1:1 mixture of eGFP-labeled S6K1 knockout cells and unlabeled wild-type cells. After five weeks, the organoids were dissociated into single cells using a Papain dissociation kit (Worthington Biochemical Corporation, #LK003150). The dissociated cells were then divided into eGFP-positive and eGFP-negative portions using a BD FACS Aria, and 50,000 sorted cells were lysed with 50 µL of nuclei isolation buffer (10 mM Tris-HCl (pH 7.4), 10 mM NaCl, 3 mM MgCl2, 0.1 % Tween-20, 0.1 % NP-40, 0.01 % Digitonin, 1 % BSA, 1 mM DTT) for 5 min on ice. Five hundred microliters of chilled wash buffer (10 mM Tris-HCl (pH 7.4), 10 mM NaCl, 3 mM MgCl_2_, 0.1 % Tween-20, 1 % BSA, and 1 mM DTT) were added to lysed cells, and cells were centrifuged at 500 × g for 5 min at 4 °C. The supernatant was removed and transposition was performed using Tn5 (Illumina). Cell pellets were resuspended in a transposition mix (2X TD buffer, 100 nM Tn5 transposase, 0.01 % digitonin, and 0.1 % Tween-20) and incubated at 37 °C (1,000 rpm) for 30 min. Fragmented DNA was purified using the MinElute reaction Cleanup kit (Qiagen, 28204) and amplified with pre-designed primers containing adaptors and barcodes. Next, the ATAC-seq libraries were purified using AMPure XP beads and the final libraries were sequenced with a paired-end read of 150 bp on the NovaSeq 6000 Illumina platform.

For analysis of ATAC-seq data, the ATAC-seq guidelines from Harvard FAS informatics were conducted. Quality control, alignment, and format transformations were the same as those described above. Peaks were called by Macs3 v3.0.0a7 through peak calling with one command [32]. The parameters were set as follows: −f BAMPE, −g hs, −B, −p 0.01, −-nomodel, −-shift 75, −-extsize 150. Peaks were combined before quantification using feature counts. The differentially enriched region test was performed using edgeR v3.38.4 (p-value < 0.01 & logFC > 2 or logFC < −2 & bcv = 0.4), and regions were annotated by CHIPseeker v1.32.0, and enriched functionally by GeneAnswers v1.16.0 (p-value < 0.05) [33,34]. Average profile plots were drawn using ngs. plot v2.63, and specific genome tracks were illustrated using Integrative Genomics Viewer (IGV) v2.13.2 [35,36].

### Statistical analysis

For RT-qPCR and quantification of images, statistical significance was analyzed using the Student’s *t*-test (two-tailed) and evaluated based on a p-value. Results with *Ρ < 0.05; **Ρ < 0.01; ***Ρ < 0.001 were considered as statistically significant.

For single-cell RNA-seq analysis, we employed the Wilcoxon Rank Sum test (via Seurat’s FindAllMarkers function) for differential gene expression analysis. This non-parametric test is widely adopted in single-cell workflows due to its robustness to zero-inflation and non-normal distributions inherent in sparse single-cell data. For bulk RNA-seq analysis, we used DESeq2, which models read counts using a negative binomial distribution to handle over dispersion. Statistical significance was assessed via Wald tests. Similarly, edgeR was applied to bulk ATAC-seq data, leveraging its negative binomial framework with empirical Bayes moderation of peak-wise dispersions.

## Results

### Deletion of S6K1 impairs brain organoid development

**Fig. 1:**
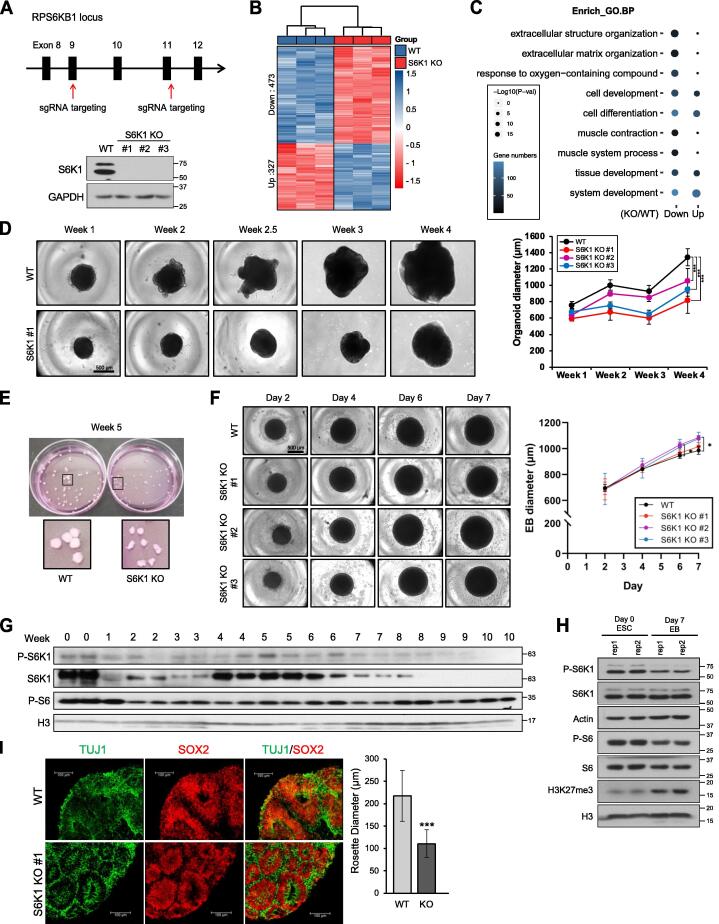
**Genetic deletion of S6K1 impairs brain organoid development. A.** Genome engineering strategy for CRISPR-based knockout (KO) of RPS6KB1 (upper). Confirmation of S6K1 deletion in H7 cells with western blotting (bottom). **B.** Heatmap of differentially expressed genes (DEGs) upon S6K1 deletion in H7 cells. **C.** Enrichment analysis of biological process terms of gene ontology (GO.BP) for DEGs upon S6K1 deletion in H7 cells. **D.** Microscopic images (left) and quantification graph for diameters (right) of embryoid bodies derived from wild-type (WT) and S6K1-/- H7 cells 1, 2, 3, and 4 weeks after starting brain organoid differentiation. E. Optical (week 5) images of dorsal forebrain organoids derived from WT and S6K1-/- H7 cells at the indicated timepoints. F. Microscopic images (left) and quantification graph for diameters (right) of embryoid bodies (EB) derived from wild-type (WT) and S6K1-/- H7 cells after 2, 4, 6, and 7 days of embryonic body formation without neuronal induction. G. Immunoblot analysis of dorsal forebrain organoids derived from WT H7 cells at the indicated timepoints. H. Immunoblot analysis of embryonic stem cell (Day 0) and embryonic body without neuronal induction (Day 7). I. Immunohistochemistry image (left) and its quantification graph (right) of TUJ1 and SOX2 in dorsal forebrain organoids derived from WT or S6K1-/- H7 cells at week 5. *P < 0.05; **P < 0.01; ***P < 0.001.

To investigate the role of S6K1 during human developmental processes, we induced CRISPR/Cas9-based S6K1 knockout in H7 human embryonic stem cells (hESCs) (Fig. 1A and Supplementary Fig. 1A). Genome-wide transcriptomic analysis of wild-type (WT) and S6K1^-/-^ hESCs (clone #1) identified 473 downregulated and 327 upregulated genes, respectively, upon S6K1 knockout (Fig. 1B). Gene ontology analysis showed that both down-regulated and up-regulated genes related to development and differentiation were highly enriched (Fig. 1C). Although the expression of the pluripotency marker (OCT4) was not significantly altered (Supplementary Fig. 1B) several genes involved in TGFβ signaling pathway were upregulated by S6K1 knockout (Supplementary Fig. 1C). Although it is hard to assert that this alteration of gene expression implies enhanced TGFβ signaling, previous literature reported that inhibition of S6K1 elevates TGFβ signaling [37]. It is known that enhanced TGFβ signaling specifies the fate of pluripotent stem cells into endoderm or mesoderm lineages, while TGFβ signaling is silenced during ectodermal differentiation (Supplementary Fig. 1D) [38,39]. Therefore, we hypothesized that S6K1 affects the ectodermal development of human pluripotent stem cells at an early stage. To examine the effects of S6K1 deletion on the development of human brain, a representative ectoderm-derived organ, we produced dorsal forebrain organoids from WT and S6K1^-/-^ hESCs based on a previously established protocol [40]. After the first week of neural induction, the size of the embryoid body was significantly reduced in all S6K1^-/-^ groups compared to that of WT group (Fig. 1D, E). During the differentiation period, enlargement of brain organoids was limited by S6K1 deletion (Fig. 1D, E). As the proliferation rate of S6K1^-/-^ hESCs was lower than that of WT hESCs (1.4-fold that of S6K1^-/-^ cells) (Supplementary Fig. 1E), we hypothesized that a lower proliferation rate might induce the microcephalic phenotype of S6K1^-/-^ organoids. Thus, we started organoid generation with 1.4-fold higher number of S6K1^-/-^ hESCs than WT hESCs at the initial step of organoid generation. However, the reduced size of organoids did not recover despite the increased number of starting cells (Supplementary Fig. 1F). Furthermore, the size of embryoid bodies grown without neural induction did not show notable reduction due to S6K1 deletion (Fig. 1F). These data suggest that S6K1 critically affects neural induction and enlargement during the initial stages of brain organoid development, accompanied by dynamic changes in S6K1 protein level, independent of the proliferation rate.

### Deletion of S6K1 reduces the size of neural rosettes in the early stage of brain organoid development

During the neural induction stage of human brain development, neuroepithelial cells first expand and transform into progenitor cells, called ventricular radial glia (vRG) [41]. Other progenitors, including outer radial glia (oRG) and mature neurons differentiated from vRG, migrate to the upper layer, and the sequential generation of deep layer and late upper layer neurons induces spatial patterns during corticogenesis [41,42]. To investigate which stage of human corticogenesis is affected by S6K1, we assessed the expression and activation levels of S6K1 during the entire developmental period of the brain organoids. Both total levels and phosphorylation of S6K1 were drastically elevated between weeks 4 and 6, whereas their signals diminished again after week 7 (Fig. 1G). Unlike embryoid bodies grown in neural induction media showing drastic reduction after 1 week (Fig. 1G), the level of S6K1 in embryoid bodies grown without neural induction remained unchanged (Fig. 1H). Previous studies showed that the expansion of oRG occurs at mid-neurogenesis [43], and mTOR specifically regulates the migration of oRG cells [44]. We also found that the expression of S6K1 was detected in the outer subventricular zone (oSVZ) layer, where HOPX, a specific marker of oRG, is expressed (Supplementary Fig. 1G, H). Immunostaining with TUJ1, a marker for the initial stage of neural differentiation, and SOX2, a ventricular zone (VZ) marker showed that neural rosettes were produced regardless of S6K1 at week 5 (Fig. 1I). However, the diameter of neural rosettes, which was measured by the SOX2-positive area, significantly decreased in S6K1^-/-^ organoids (Supplementary Fig. 1H). This phenotypic change, reduced rosette size caused by S6K1 deletion, apparently leads to the microcephaly observed during early brain organoid development (Fig. 1D, E).

### Abnormal emergence of retinal lineage cells in S6K1-deleted brain organoid at week 5

**Fig. 2:**
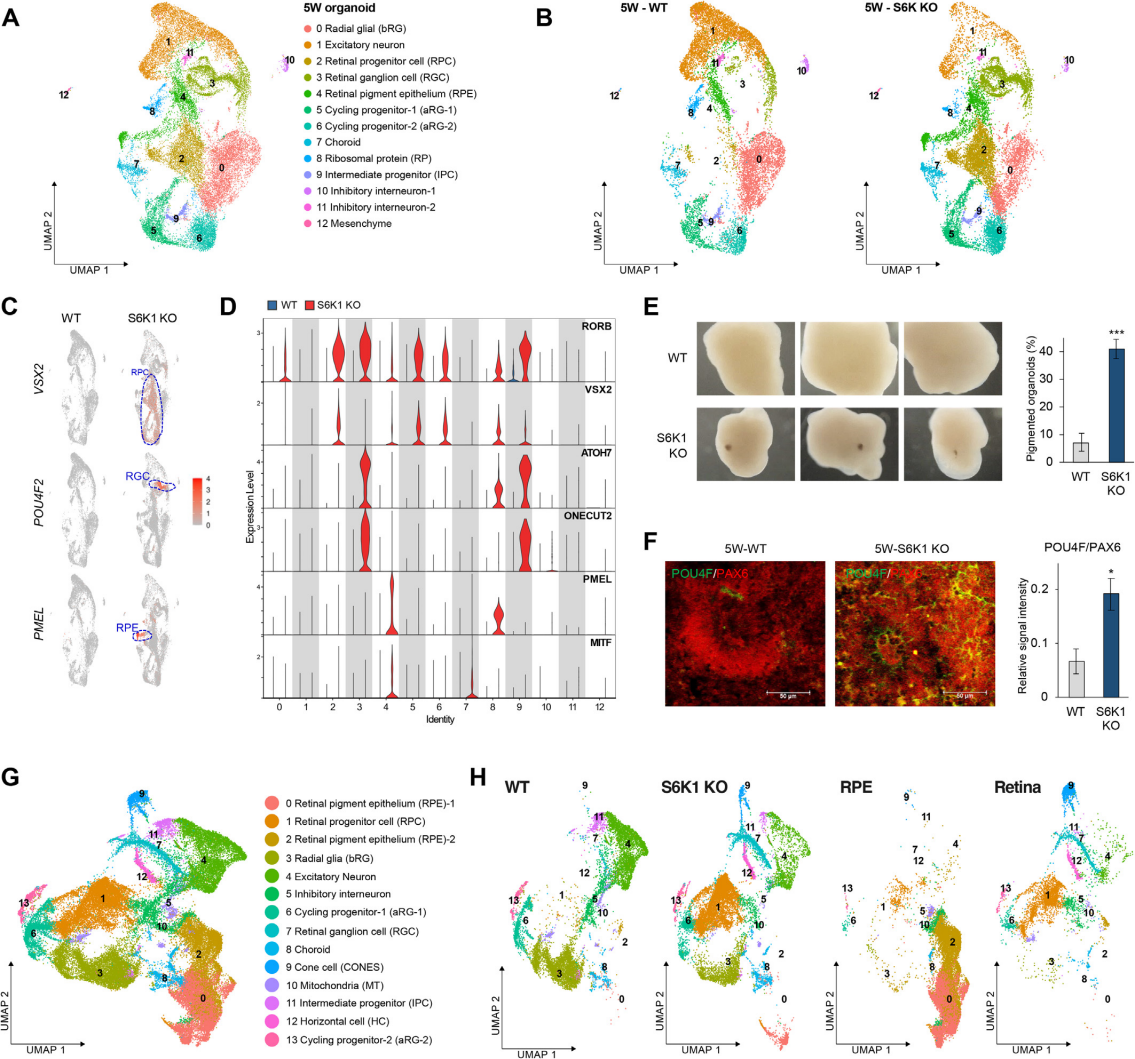
**Abnormal emergence of retinal lineage cells in S6K1-deleted brain organoids. A.** Integrated UMAP and cluster annotation of single cell RNA sequencing with dorsal forebrain organoids derived from WT and S6K1^-/-^ H7 cells at week 5. Duplicates for each group (5 ∼ 10 organoids for each batch) were subjected to single cell RNA sequencing. 6015 and 9782 cells for duplicates of WT were analyzed, and 7423 and 7140 cells for duplicates of S6K1^-/-^ were analyzed. **B.** Separated UMAPs of the dorsal forebrain organoids derived from WT (left) or S6K1 KO (right) cells at week 5. **C.** Feature plots showing the expression of retinal lineage marker genes. **D.** Violin plots showing relative expression of retinal lineage marker genes across clusters. **E.** Microscopic images of dorsal forebrain organoids derived from WT and S6K1^-/-^ H7 cells at week 5. Quantification graph shows mean ± SD (3 batches with 6 ∼ 12 organoids/batch) **F.** Immunohistochemistry images of dorsal forebrain organoids derived from WT and S6K1^-/-^ H7 cells at week 5. Signal intensities of POU4F1 were normalized to PAX6 signal intensities. Mean ± SD (n = 3). **G.** Integrated UMAP and cluster annotation of single cell RNA sequencing with dorsal forebrain organoids derived from WT and S6K1^-/-^ H7 cells at 5 weeks, human RPE, and human retina. **H.** Separated UMAPs of dorsal forebrain organoids derived from WT, S6K1^-/-^ cells, RPE, and retina. *P < 0.05; **P < 0.01; ***P < 0.001.

To investigate the differences between WT and S6K1^-/-^ brain organoids at the single-cell level, we performed single-cell RNA sequencing (scRNA-seq) at the five weeks after organoid generation (Fig. 2A). Thirteen clusters were identified and annotated based on cluster-specific gene expression patterns (Fig. 2A, Supplementary Fig. 2A, B). To explore fates of the cycling progenitor cells, the earliest cell types committed from pluripotent state, we extracted clusters 5 and 6 (cycling progenitor-1 and 2, respectively) and detected marker genes for proliferation (ASPM, TOP2A, CCNA1, and CDC25B), apoptosis (BAX, BCL21, GADD45A, and XIAP), ectoderm differentiation (SOX2 and PAX6), and telencephalic region (FOXG1 and EMX1) in the feature plots (Supplementary Fig. 2C-F). Whereas proliferation markers expressed slightly more in cluster 5 than cluster 6 (Supplementary Fig. 2C), apoptotic markers mingled in both clusters (Supplementary Fig. 2D). Interestingly, while ectodermal differentiation markers were expressed in most cells without large difference between WT and S6K1 KO organoids (Supplementary Fig. 2E), telencephalic markers showed stronger signal in WT than S6K1 KO organoids (Supplementary Fig. 2F). These data imply the lineage specification toward non-telencephalic ectodermal cells in S6K1 KO organoids. In line with this notion, three clusters (clusters 2, 3, and 4) related to retinal cell lineages were specifically observed in S6K1^-/-^ brain organoids (Fig. 2B). Genes specifically expressed in clusters 2, 3, and 4 were extracted and subjected to Enrichr, which contains a large collection of gene set libraries [45]. Comprehensive analysis of a publicly available data (Jensen TISSUES and Human Gene Atlas) in Enrichr showed that genes of the three clusters were closely related to the eye and retina (Supplementary Fig. 3A). By analyzing the expression patterns of marker genes of each retinal sub-cluster (retinal progenitor cells [RPC], retinal ganglion cells [RGC], and retinal pigment epithelium [RPE]), we confirmed that their expression was predominantly detected in S6K1^-/-^ brain organoids (Fig. 2C). Violin plots also indicated that retinal lineage marker genes were mainly expressed in S6K1^-/-^ brain organoids but were hardly detected in WT brain organoids (Fig. 2D). Consistent with the emergence of RPE in S6K1^-/-^ brain organoids, pigmented areas were more frequently observed in S6K1^-/-^ brain organoids than in WT brain organoids (Fig. 2E). Moreover, immunostaining data showed that the expression of POU4F, a human retinal marker, was significantly higher in S6K1^-/-^ brain organoids than in WT brain organoids upon normalization to the signal of PAX6 that is co-expressed in both retinal progenitor and neural progenitor cells (Fig. 2F). In our previous study, we revealed that S6K1 can induce H3K27me3 by promoting the recruitment of EZH2, an enzymatic subunit of the polycomb repressive complex 2 (PRC2) [2]. Therefore, we investigated whether this epigenetic function of S6K1 is involved in lineage specification between retinal and neural lineages. Comprehensive analysis of public data from the ENCODE project identified that a large portion of the genes specifically expressed in clusters 2, 3, and 4 was under the control of the PRC2 components, SUZ12 and EZH2 (Supplementary Fig. 3B). As expected, retinal cluster-specific genes were highly related to H3K27me3 (Supplementary Fig. 3C), which provides mechanistic evidence for the transcriptional regulation of abnormal lineage specification in S6K1^-/-^ brain organoids.

To distinguish whether the cell types of clusters 2, 3, and 4 are retinal lineage cells or neural progenitors expressing retinal markers, we conducted combined analysis of our single cell RNA seq data displayed in Fig. 2A-D with public single cell RNA seq data of human RPE and human retina tissues [46,47]. Fourteen clusters were annotated based on cluster-specific gene expression patterns (Fig. 2G, Supplementary Fig. 4A, B). As expected, clusters 1 (retinal progenitor cell), 7 (retinal ganglion cell), 9 (cone cell), and 12 (horizontal cell) are largely overlapped in S6K1 KO organoids and retina (Fig. 2H) with retinal lineage marker expression in those two samples (Supplementary Fig. 4C). However, only a few cell populations expressing RPE markers in S6K1 KO overlapped with RPE, and WT brain organoids didn’t show much overlap with either RPE or retina (Fig. 2H, Supplementary Fig. 4C). WT organoids robustly generate the forebrain lineage predominantly expressing canonical telencephalic markers (FOXG1 and EMX1), while non-telencephalic markers (OTX2 and WLS) were detected in choroid and retinal lineage cells (Supplementary Fig. 4D). These combined analysis data suggest that limited portion of RPE cells and relatively more portion of retinal cells were developed in S6K1 KO brain organoids.

### Mature neuron populations are reduced in S6K1-deleted brain organoid at week 5

**Fig. 3:**
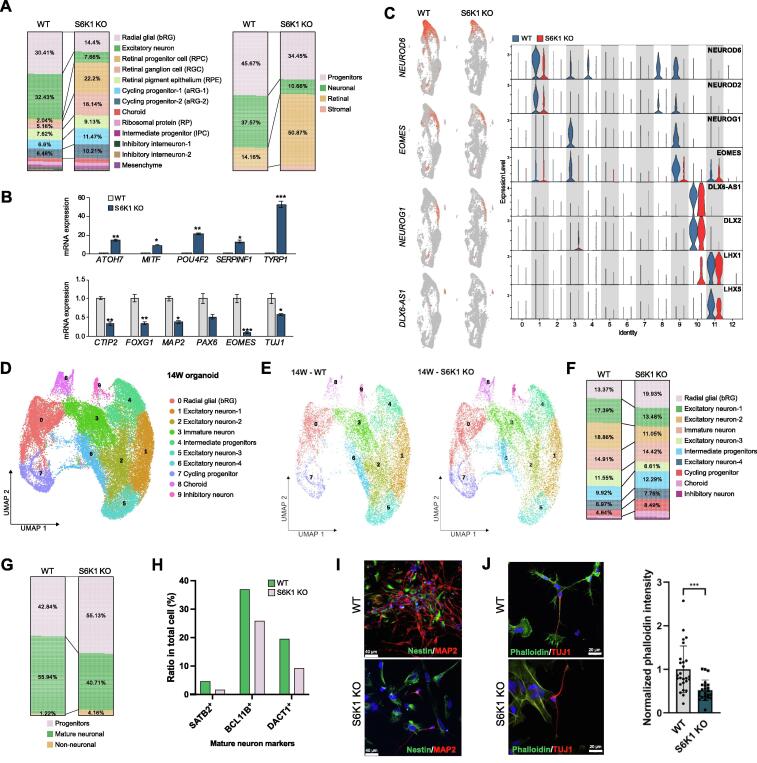
**S6K1 deletion reduces mature cortical neurons in brain organoids. A.** Portion of each cluster (left) and progenitor (cluster 0, 5, 6, and 9), neuronal (cluster 1, 10, and 11), retinal (cluster 2, 3, and 4), and stromal (cluster 7, 8, and 12) cell clusters (right) in single cell RNA sequencing data of dorsal forebrain organoids derived from WT and S6K1^-/-^ H7 cells at week 5. **B.** The mRNA levels of retinal (upper) and neuronal (bottom) marker genes were analyzed through bulk RT-qPCR of dorsal forebrain organoids derived from WT and S6K1^-/-^ H7 cells at week 5. Mean ± SEM (n = 3 or 4). **C.** Feature plots (left) and violin plots (right) showing the relative expression of neuronal lineage marker genes across clusters. **D.** Integrated UMAP and cluster annotation of single cell RNA sequencing with dorsal forebrain organoids derived from WT and S6K1^-/-^ H7 cells at week 14. Duplicates for each group (2 ∼ 3 organoids for each batch) were subjected to single cell RNA sequencing. 9,150 and 10,240 cells for duplicates of WT were analyzed, and 9,722 and 9,617 cells for duplicates of S6K1^-/-^ were analyzed. **E.** Separated UMAPs of dorsal forebrain organoids derived from WT (left) or S6K1^-/-^ (right) cells at week 14. **F, G.** Portion of each cluster (F) and progenitor (cluster 0, 3, 5, and 7), mature neuronal (cluster 1, 2, 4, 6, and 9), and non-neuronal (cluster 8) cell clusters (G) in single cell RNA sequencing data of dorsal forebrain organoids derived from WT and S6K1^-/-^ H7 cells at week 14. **H.** The percentage of cells expressing indicated markers among all cells in single cell RNA sequencing data of dorsal forebrain organoids derived from WT and S6K1^-/-^ H7 cells at week 14. **I, J.** Immunocytochemistry of monolayered cortical neurons differentiated from WT and S6K1^-/-^ cells at day 37. Graph indicates the phalloidin intensity in TUJ1-positive cells. Mean ± SD (n = 26 for WT and 23 for S6K1 KO). *P < 0.05; **P < 0.01; ***P < 0.001.

Cell population analysis of scRNA-seq data with 5 weeks-old organoids showed that the proportion of retinal lineage cells (clusters 2, 3, and 4) was markedly increased in S6K1^-/-^ brain organoids compared to that of WT brain organoids, while the population of neuronal lineage cells (clusters 1, 10, and 11) was reduced in S6K1^-/-^ brain organoids (Fig. 3A). Bulk RT-qPCR of 5 weeks organoids also indicated that the expression of retinal marker genes was enhanced in S6K1^-/-^ brain organoids (Fig. 3B, upper). In contrast, the expression of multiple neural marker genes in organoids decreased upon S6K1 deletion (Fig. 3B, bottom). This tendency was confirmed using two additional S6K1 KO cell lines (Supplementary Fig. 4E) and six additional biological replicates (Supplementary Fig. 4F), eliminating the possibility of clonal differences and batch effects due to technical variability. Consistently, as shown in the UMAP and violin plots, the expression of excitatory neuron-specific genes in multiple neural populations was higher in WT brain organoids than that of S6K1^-/-^ brain organoids (Fig. 3C). Interestingly, the marker genes of inhibitory neurons, which are minor portions of dorsal forebrain organoids, did not show significant differences between them (Fig. 3C).

In Cluster 9, which was annotated as intermediate progenitor cells (IPCs), WT and S6K1^-/-^ brain organoids showed distinct genetic patterns; retinal marker genes were mainly expressed (Fig. 2D) in S6K1^-/-^, and IPC marker genes were mainly detected in WT (Fig. 3C). As ganglion cells are a type of neuron developed by retinal neurogenesis from multipotent retinal progenitor cells [48], it is assumed that some RGC populations in S6K1^-/-^ brain organoids were grouped into the same cluster as the IPC of WT brain organoids. Collectively, both population of cortical neurons and the expression of neural lineage cells were downregulated in S6K1 KO organoids at the mid-stage development (week 5).

### Mature neuron populations are reduced in S6K1-deleted brain organoid at week 14

Next, we performed scRNA-seq with mature brain organoids grown for 14 weeks to investigate whether retinal lineage cells remained in the in S6K1^-/-^ brain organoids (Fig. 3D). Ten clusters were annotated based on cluster-specific gene expression patterns (Fig. 3D, Supplementary Fig. 5A, B). Interestingly, retinal cell populations were not identified in either WT or S6K1^-/-^ brain organoids (Fig. 3D, E, Supplementary Fig. 5C). Without essential morphogens for retinal lineage differentiation in growing media, the retinal lineage cells observed at 5 weeks apparently failed to go through further development. In addition, the proportion of mature neurons (clusters 1, 2, 5, 6, and 9) decreased in S6K1^-/-^ brain organoids, whereas the neural progenitor cell portion (clusters 0, 3, 4, and 7) was higher in S6K1^-/-^ brain organoids compared to that of WT brain organoids (Fig. 3F, G). To compare further maturation of neuronal cells in WT S6K1^-/-^ brain organoids, we evaluated the ratio of cells expressing SATB2, an upper-layer neuron marker [49], CTIP2/BCL11B, a deep-layer neuron marker [50], and DACT1, a postsynaptic protein [51]. Portion of cells expressing the marker of functionally mature neurons (SATB2, BCL11B/CTIP2, and DACT1) were decreased in S6K1^-/-^ brain organoids (Fig. 3H). Considering that the portion of neural progenitors of S6K1^-/-^ brain organoids was lower than WT at mid-term development (Fig. 3A), the increased progenitor portion and decreased mature neuron portion by S6K1 deletion in late-stage development imply the incomplete maturation of neural stem cells into mature neurons.

To compare the morphology of cells within organoids, we dissociated brain organoids grown for 5 and 14 weeks and then replated the dissociated cells as monolayer. In microscopic images, the isolated cells from WT brain organoids showed long and fine dendrite-like organelles branched from the cell body, which is a typical morphology of mature neurons (Supplementary Fig. 5D). However, many of the cells dissociated from S6K1^-/-^ brain organoids showed larger cell bodies without dendrites, representing the immature neuronal population of S6K1^-/-^ brain organoids (Supplementary Fig. 5D). Next, we generated 2-dimensional (2D) cortical neurons from WT and S6K1^-/-^ hESCs, which showed morphological features and cell body size distribution similar to those of organoid-derived 2D neurons (Supplementary Fig. 5E). The immunostaining of cortical neurons at the mature stage (day 37) identified that the expression of MAP2, a mature neuron marker, was reduced in the S6K1^-/-^ group, while the expression of nestin, a neural lineage cell marker, was similar in WT and S6K1^-/-^ neurons (Fig. 3I). Previous studies demonstrated that the expression of both filamentous (F)-actin and microtubules (MTs), cytoskeletal polymers, is required for construction of growth cone and axon outgrowth of neurons. In addition, acetylation of alpha-tubulin is necessary for stable microtubule bundles and neurite extension of neuron [[52], [53], [54]]. Immunostaining with phalloidin to detect F-actin showed that the construction of a growth cone was impaired in S6K1^-/-^ neurons compared to WT neurons (Fig. 3J). The acetylated tubulin signals were also diminished in S6K1^-/-^ neurons, consistent with impaired axon outgrowth of neurons in S6K1^-/-^ group (Supplementary Fig. 5F).

Collectively, the data displayed in Fig. 3 demonstrate that S6K1 is required for the further maturation of neural stem cells into mature neurons during the late stage of brain organoid development.

### Dual specification into retinal lineage and cortical neuron of S6K1-deleted brain organoid

**Fig. 4:**
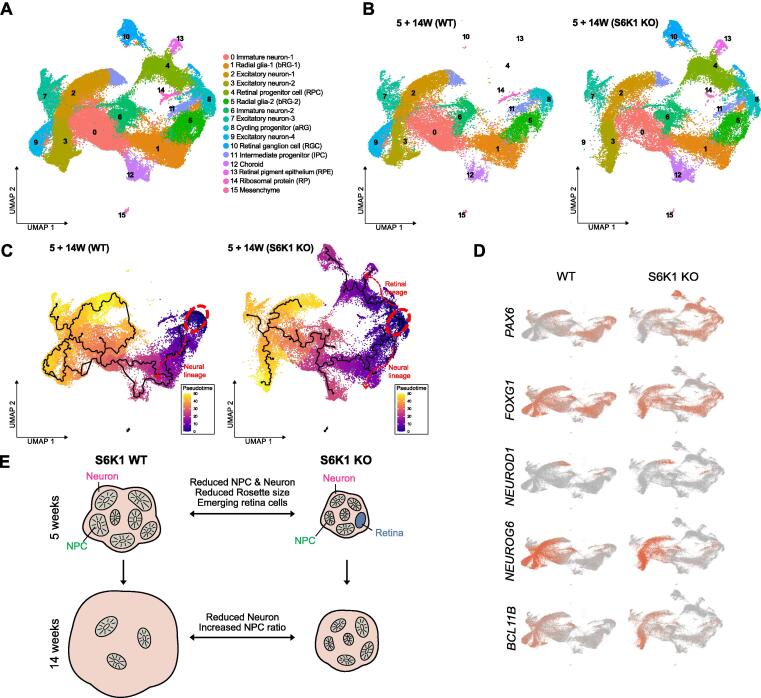
**Dual specification into retinal lineage and cortical neuron during brain organoid development by S6K1 deletion. A.** Integrated UMAP and cluster annotation of single cell RNA sequencing with dorsal forebrain organoids derived from WT and S6K1^-/-^ H7 cells at week 5 and 14. **B.** Separated UMAPs of dorsal forebrain organoids derived from WT and S6K1^-/-^ H7 cells at week 5 and 14. **C.** Pseudo time analysis of dorsal forebrain organoids derived from WT and S6K1^-/-^ H7 cells at weeks 5 and 14. **D.** Feature plots showing the relative expression of neuronal lineage marker genes. **E.** Schematic representation demonstrating the outcomes of the dysregulated mTOR/S6K1 signaling pathway during human brain development.

To compare the developmental timeline, we integrated the scRNA-seq data from organoids grown for 5 and 14 weeks and performed a pseudotime analysis. In combined UMAP, 16 clusters were identified, and retinal lineage cell populations (clusters 4, 10, and 13) were specifically observed in S6K1^-/-^ brain organoids (Fig. 4A, B). To infer the trajectories of the populations, cluster 8 (cycling progenitor) was designated as the starting population (Fig. 4C). In WT brain organoids, the starting population were clearly differentiated into a neural lineage through neural progenitors and mature excitatory neurons in which diverse neuronal markers were detected (Fig. 4C, left, D). However, S6K1^-/-^ brain organoids showed dual lineage specification into neural and retinal lineages, as divergence of the two lineages occurred when cluster 8 cells developed into cluster 5 (radial glia) and cluster 4 (retinal progenitor cells) (Fig. 4C, right) and PAX6, a dual marker for retinal progenitor and neural progenitor, was detected in retinal lineage as well as neuronal lineage (Fig. 4D). Taken together, our data showed that deletion of S6K1 during the entire period of brain development induced abnormal phenotypes in multiple stages (Fig. 4E). Microcephaly and abnormal emergence of retinal lineage cells with reduced neural lineage differentiation were observed in early stage (∼5 weeks), while reduced portion of mature neurons accompanied by accumulated neural progenitors was exhibited in late stage (Fig. 4E).

### Retinal emergence in S6K1-deleted brain organoids results from non-cell-autonomous action

**Fig. 5:**
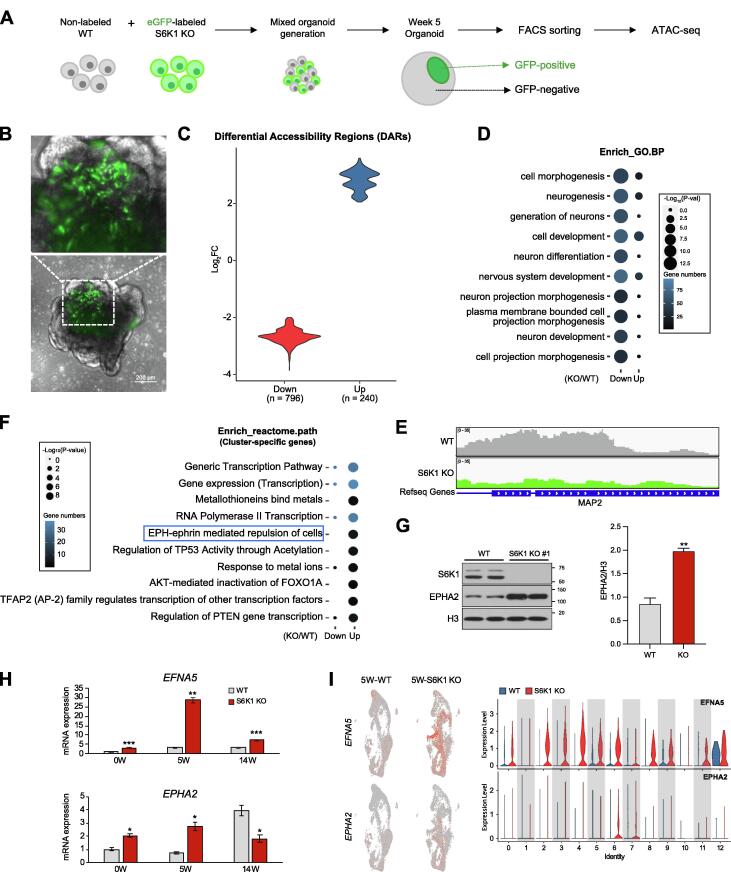
**Ephrin signaling-mediated non-cell autonomous action of S6K1 during brain development. A.** Experimental scheme for ATAC sequencing of WT and S6K1^-/-^ cells from mixed organoid. **B.** Microscopic images of dorsal forebrain organoids derived from mixed cell populations of non-labeled WT and GFP-labeled S6K1^-/-^ cells. **C.** Violin plot showing the differentially enriched regions in the GFP-positive (S6K1^-/-^) population versus GFP-negative (WT) population. **D.** Enrichment analysis of biological process terms of gene ontology (GO.BP) for up/down-regulated genes in the GFP-positive (S6K1^-/-^) population versus GFP-negative (WT) population. **E.** IGV track of ATAC-sequencing signals on the MAP2 gene region. **F.** Enrichment analysis of the Reactome Pathway (reactome.path) for up/down-regulated genes upon S6K1 deletion in H7 cells. **G.** Immunoblot analysis of dorsal forebrain organoids derived from WT and S6K1^-/-^ H7 cells at week 5. Quantification graph indicates mean ± SD (n = 3). **H.** The mRNA levels of EFNA5 and EPHA2 were analyzed through bulk RT-qPCR of the dorsal forebrain organoids derived from WT and S6K1^-/-^ H7 cells at week 0, 5, and 14. Mean ± SEM (n = 3 or 4). *P < 0.05; **P < 0.01; ***P < 0.001. **I.** Feature plots (left) and violin plots (right) showing the relative expression of EFNA5 and EPHA2 across clusters in single cell RNA sequencing analysis with dorsal forebrain organoids derived from WT and S6K1^-/-^ H7 cells at week 5.

Previous studies suggest that the segregation of cells with similar adhesive properties contributes to spatially distinguished cell-fate decisions and stably patterned organogenesis at the early stage of tissue development [55,56]. To investigate whether the positional identity of the retina within the brain depends on S6K1 activity, we generated brain organoids with mixed cell populations of WT and S6K1^-/-^ hESCs (Fig. 5A). Before mixing the cells, S6K1^-/-^ hESCs were infected with GFP. At week 5, the organoids of mixed cell populations were dissociated and sorted into GFP-positive and GFP-negative cells, followed by ATAC-sequencing of these two portions (Fig. 5A). Interestingly, the sorting phenomenon was observed in mixed organoids showing positional segregation of GFP-positive S6K1^-/-^ cells from GFP-negative WT cells (Fig. 5B), which was not displayed in undifferentiated embryoid bodies (Supplementary Fig. 6A). Moreover, the ATAC sequencing identified differentially regulated genomic regions (Fig. 5C). Gene ontology analysis showed that downregulated genomic regions in GFP-positive S6K1^-/-^ cells were highly enriched in neural differentiation/development-related GO terms (Fig. 5D). The IGV genome browser view of MAP2, a representative neural lineage marker gene, showed extensively decreased chromatin accessibility in GFP-positive S6K1^-/-^ cells compared to GFP-negative WT cells (Fig. 5E). Unexpectedly, the chromatin accessibility of retinal cluster-specific genes identified in scRNA-seq data of Fig. 3 showed similar patterns in GFP-positive S6K1^-/-^ and GFP-negative WT portions (Supplementary Fig. 6B, C). These data suggest that S6K1 has both non-cell-autonomous function for the suppression of retinal fate determination and cell-autonomous function for the expression of neural lineage marker genes. Based on these findings, we hypothesized that dual lineage specification within S6K1^-/-^ brain organoids would be mediated by non-cell autonomous action.

The boundaries of specific tissues within the embryo are established by the balance between cell–cell repulsion by Ephrin-Eph receptor pairs and cadherin-mediated cell adhesion [57]. In particular, the interaction between Ephs and Ephrins induces segregation of the eye field from neural territories during the early stages of forebrain morphogenesis [58]. GO analysis of our bulk RNA-seq data (Fig. 1B) showed that the up-regulated genes in S6K1^-/-^ hESCs were enriched in EPH-ephrin signal pathway (Fig. 5F). Thus, we checked the expression of ephrin-A5 (EFNA5) and ephrin type-A receptor (EPHA2), essential genes for lens development and patterning of lens cells [59,60], in WT and S6K1^-/-^ brain organoids. The higher expression of EPHA2 was detected in S6K1^-/-^ brain organoids compared to WT brain organoids (Fig. 5G). The RNA expression levels of both EFNA5 and EPHA2 were also increased in S6K1^-/-^ organoids, especially in retinal cell clusters, compared to WT brain organoids (Fig. 5H, I).

In addition to ephrin signaling-dependent cell repulsion, strong adhesion between eye cells contributes to eye field segregation, making eye cells resistant to neurulation movements [61]. The embryonic ectoderm initially expresses E-cadherin, which is replaced by N-cadherin upon neurulation [62]. The other previous studies also showed that collagen disrupts E-cadherin-mediated cell–cell adhesion [63,64]. At the embryonic stage, S6K1 knockout reduced the expression of gene sets related with collagen-containing extracellular matrix organization (Supplementary Fig. 6D-F). Moreover, E-cadherin was more abundant in S6K1^-/-^ hESCs than in WT ESCs (Supplementary Fig. 6G). Interestingly, in 5 weeks-old organoids that highly expressed N-cadherin instead of E-cadherin (Supplementary Fig. 6H), S6K1^-/-^ cells showed higher expression of N-cadherin than WT (Supplementary Fig. 6G). Considering that the EPHA2-EFNA5 interaction enhances the binding of β-catenin to N-cadherin [59], the cooperative action of Ephrin signaling and adhesion molecules might be one possible driver which allow S6K1^-/-^ cells to differentiate into retinal cells (Supplementary Fig. 6I).

## Discussion

Although the effects of hyperactive mTOR/S6K1 signaling on the human brain have been recurrently investigated, this is the first study to reveal the influence of constitutively inactive S6K1 during human brain development. Microscopic images and immunostaining revealed microcephaly and smaller neural rosettes in early stage of S6K1-deleted brain organoids. Although cycling progenitor and radial glia populations were clearly distinguished in scRNA-seq data and immunostaining exhibiting initial differentiation forming cortical layers, pseudotime analysis of scRNA-seq data predicted that retinal progenitors diverge from apical radial glia (aRG), which is parallel with normal differentiation of basal radial glia (bRG) from aRG. Therefore, abnormal dual specification in S6K1^-/-^ brain organoids starts when cycling progenitor/aRG generate the second niche, bRG.

Even though we used a protocol to induce guided differentiation into the dorsal forebrain, which known to have limited retinal cell specification, spontaneous emergence of retinal lineage cells was observed in S6K1-deleted organoids. As corticogenesis and retinogenesis exhibit similar mechanisms of differentiation and maturation [65], it has been difficult to discover which signaling molecules are critically responsible for the acquisition of distinct cell fates. Our present findings reveal that S6K1 is required at the branching point to suppress retinogenesis and commit progenitors to cortical neurons. The impaired neural specification caused by S6K1 deletion stems from both cell- and non-cell-autonomous actions. Retinal emergence might be induced by enhanced cell repulsion and adhesion, which are critical for eye field establishment. Despite the significance of cell adhesion and repulsion in the construction of optic vesicles, the precise mechanism by which self-patterning of the eye field occurs in the early stage without spatial topography has been poorly understood. In a recent study for the assembled optic vesicle-containing brain organoids (OVB organoids), the brain organoids were not treated with any morphogen or drug in the initial stage but depended on the intrinsic ability to constitute the sensory structures [66]. Our current attempt to determine the spatial gradient of S6K1 by mixing embryonic stem cells with different genetic traits failed to induce retinal development. Although retinal cell types were observed in homogenous S6K1^-/-^ brain organoids, these populations did not mature enough to develop functional optic cells. Taken together, inactivation of S6K1 is required only in the initial stage of visual system development by altering cell–cell interaction, but not during further maturation of primitive retinal cells.

Intriguingly, abnormal emergence of the retinal population was observed in dorsal forebrain organoids from chimpanzee iPSCs [10]. The mTOR signaling pathway is active in human oRG cells residing in the oSVZ layer but weaker in non-human primate brain organoids [10].This human-specific feature contributes to evolutionary changes, although the overall developmental process is conserved across species [10,44]. In addition, inactivation of mTOR/S6K1 can explain retinal development in chimpanzee brain organoids, which was dismissed as an accidental off-target lineage specification [10].

In addition to the evolutionary expansion of the brain, constitutive hyperactivation of mTOR during human brain development is associated with diverse neurological symptoms. Mosaicism caused by somatic mutations in MTOR leads to malformation of the cerebral cortex, disrupted neuronal migration, and epileptic seizures [67,68]. ATAC-seq data of organoids from mixed cells showed that impaired neural development due to S6K1 knockout is a cell-autonomous action that depends on the genetic traits of each cell. Heterozygous germline mutations in MTOR cause Smith-Kingsmore syndrome, which is characterized by macrocephaly, intellectual disability, and seizures [69]. Together with our current observations, on/off switching of mTOR/S6K1 activity at appropriate times can fine-tune human brain development, and dysregulated mTOR/S6K1 signaling induces brain anomalies.

However, some key questions remain unanswered and open for further investigation. First, the intrinsic mechanism underlying the reduced expression of neuronal marker genes in S6K1^-/-^ brain organoids must be identified to accurately understand the cell-autonomous action of S6K1 during neurogenesis. Another question is whether ephrin signaling is involved in the epigenetic control of retinal cell marker genes. Although many retinal cell cluster-specific genes are under the control of the suppressive histone modulator PRC2-H3K27me3 (Supplementary Fig. 2), experimental evidence explaining the effects of increased ephrin signaling on dysregulated epigenetic regulation is limited. Nevertheless, our current findings demonstrate that S6K1 signaling is essential for cell fate specification in cortical neurons during the early stages of human brain development by restricting differentiation into retinal progenitors.

## Conclusion

In summary, we elucidated that S6K1 deletion during human brain organoid development drives abnormal development of retinal lineage cells as well as microcephaly and reduced mature neurons. In addition to the footprint of retinal-neuronal divergence, S6K1 can be highlighted as an important molecule related to the evolutionary features of the human brain and clinical outcomes of mTOR-mutated diseases.

## Data availability

All sequencing data have been deposited in GEO (GSE214341) and will be publicly available from the date of publication. The codes used for processing the data reported in this study are available upon request from the corresponding authors.

## Declaration of competing interest

The authors declare that they have no known financial interests or personal relationships that could have appeared to influence the work reported in this paper.
