# Supplementary material for: Divergence between neural and retinal lineage specification during human brain development by signal transduction
Source: J Adv Res. 2025 Oct 22;85:375–88. doi: 10.1016/j.jare.2025.10.034 (PMC13316595; doi:10.1016/j.jare.2025.10.034)
Supplement: Supplementary Data 1 [file mmc1.docx]

**Table S1: List of RT-qPCR primers in this study**.

| Gene | Forward Sequence (5’-3’) | Reverse Sequence (5’-3’) |
| --- | --- | --- |
| ATOH7 | TTTATTCGCATCATCAGACC | CAATCAACCCATTCACAAGA |
| MITF | TTCACGAGCGTCCTGTATGCAGAT | TTGCAAAGCAGGATCCATCAAGCC |
| POU4F2 | AAGCCTACTTTGCCATTC | GCTCCCTCTTCAGTCCTC |
| SERPINF1 | TATGACCTGTACCGGGTGCGAT | CCACACTGAGAGGAGACAGGAGC |
| TYRP1 | CCGAAACACAGTGGAAGGTT | TCTGTGAAGGTGTGCAGGA |
| CTIP2 | TCCAGAGCAATCTCATCGTG | GCATGTGCGTCTTCATGTG |
| FOXG1 | AGGAGGGCGAGAAGAAGAAC | TCACGAAGCACTTGTTGAGG |
| MAP2 | CAGGAGACAGAGATGAGAATTCC | CAGGAGTGATGGCAGTAGAC |
| PAX6 | GTGTCCAACGGATGTGTGAG | CTAGCCAGGTTGCGAAGAAC |
| EOMES | AGCCGACAATAACATGCAGGG | TCCTGTCTCATCCAGTGGGA |
| TUJ1 | TGATGAACATGGCATCGAC | TATTTGCCACCTGTGGCTTC |
| EFNA5 | TGGAGCGCCAGTGTGCTGTG | AGCCCCTGCGGTCAAGGAGT |
| EPHA2 | CAAGATTGACACCATTGCGC | TCGAAGTCGCTGCTGACG |
| CDH1 | GGTTATTCCTCCCATCAGCT | CTTGGCTGAGGATGGTGTA |
| CDH2 | GCGTCTGTAGAGGCTTCTGG | GCCACTTGCCACTTTTCCTG |
