# Supplementary material for: Divergence between neural and retinal lineage specification during human brain development by signal transduction
Source: J Adv Res. 2025 Oct 22;85:375–88. doi: 10.1016/j.jare.2025.10.034 (PMC13316595; doi:10.1016/j.jare.2025.10.034)
Supplement: Supplementary Data 2 [file mmc2.pdf]

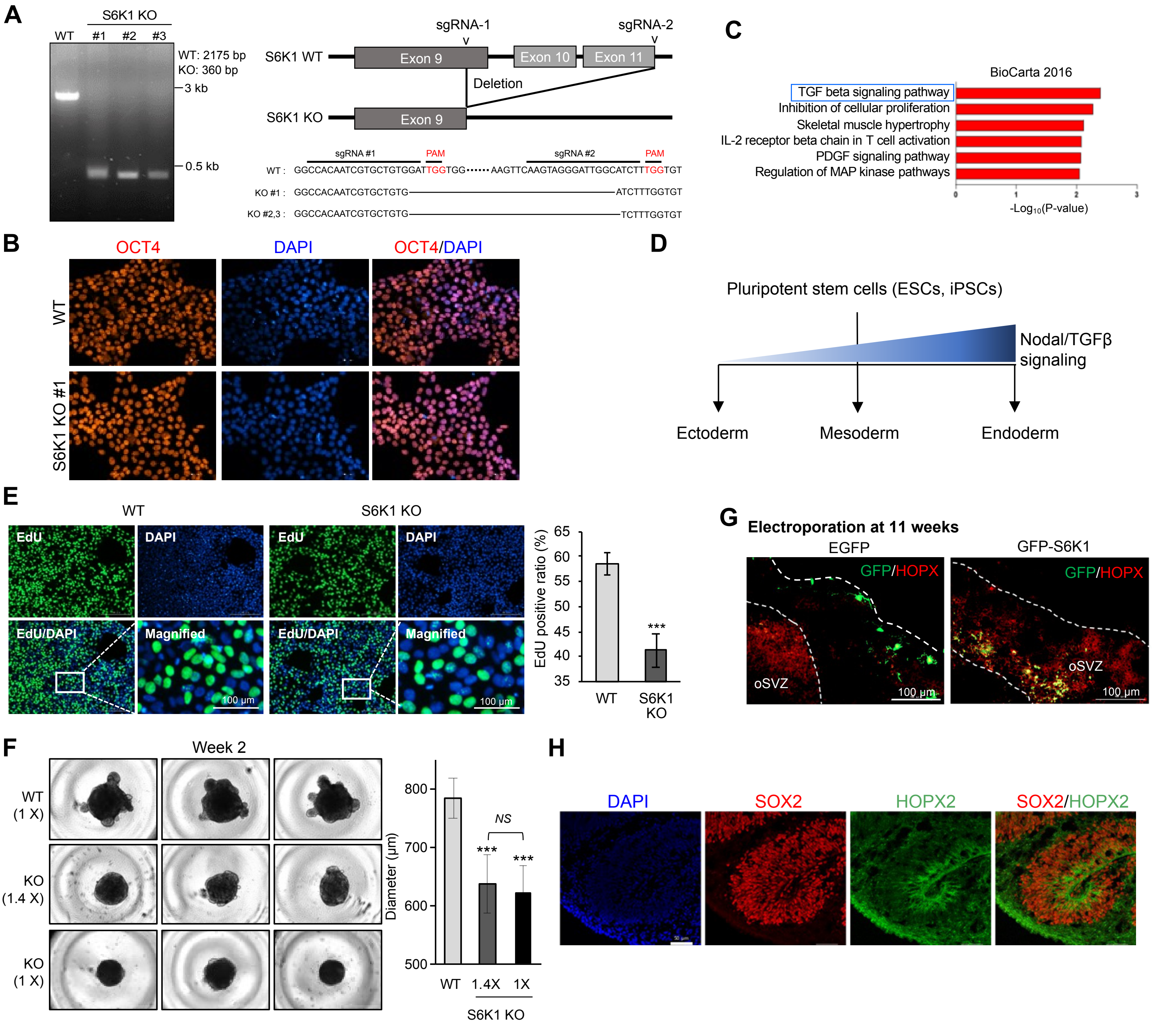

Fig. S1. S6K1 deletion impairs brain organoid development inducing dyslamination of outer radial glia.
(A) PCR genotyping for confirmation of S6K1 deletion in 3 different clones of H7 cells.
(B) Immunocytochemistry of OCT4 and DAPI in wild-type (WT) and S6K1 knockout (KO) H7 cells.
(C) Enrichment analysis of BioCarta 2016 by EnrichR for significantly upregulated genes in S6K1 KO H7 cells.
(D) Schematic representation of cell fate determination of pluripotent stem cells by Nodal/TGFβ signaling.
(E) EdU staining for detection of cell proliferation in WT and S6K1 KO H7 cells (left). Proliferation rate was calculated by EdU positive cell ratio (right).
(F) Microscopic images of embryoid bodies derived from wild-type (WT) and S6K1 KO H7 cells 2 weeks after starting brain organoid differentiation (left). 1.4 fold more cells (1.4 X) and same number of cells (1 X) were used for S6K1 KO groups. The diameter of embryoid bodies were quantified (right).
(G) Immunofluorescence images of dorsal forebrain organoids derived from WT H7 cells after transfected with EGFP or GFP-S6K1 plasmid at week 11.
(H) Immunohistochemistry of DAPI, SOX2, and HOPX2 in dorsal forebrain organoids derived from WT H7 cells at week 5.
