# Supplementary material for: Divergence between neural and retinal lineage specification during human brain development by signal transduction
Source: J Adv Res. 2025 Oct 22;85:375–88. doi: 10.1016/j.jare.2025.10.034 (PMC13316595; doi:10.1016/j.jare.2025.10.034)
Supplement: Supplementary Data 3 [file mmc3.pdf]

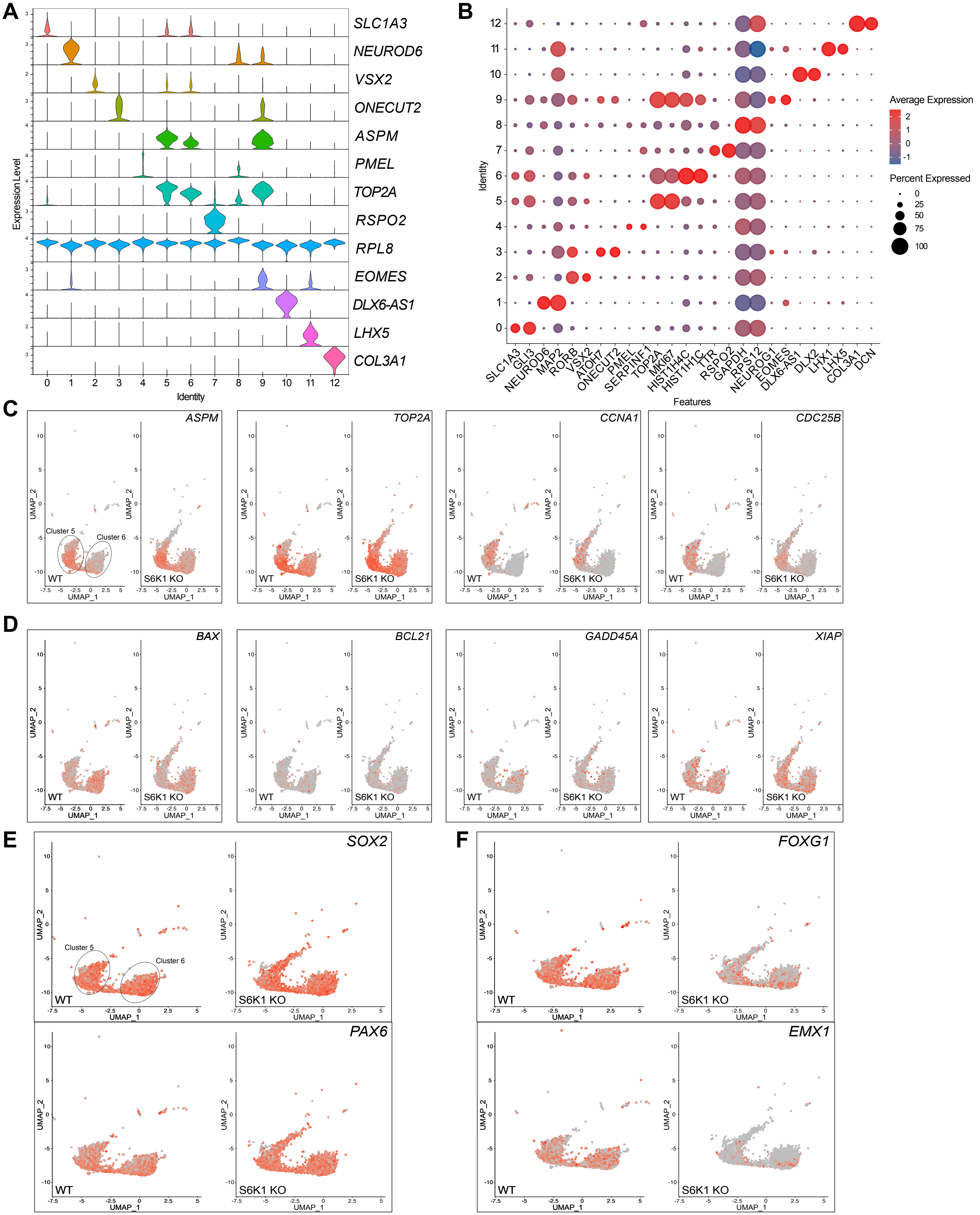

Fig. S2. Transcriptome analysis of S6K1-depleted dorsal forebrain organoids grown up to 5 weeks at the single-cell level.
(A) Violin plot showing relative expression of differentially expressed genes for each cluster in single cell RNA sequencing analysis at week 5.
(B) Dot plot showing relative expression of differentially expressed genes across clusters in single cell RNA sequencing analysis at week 5.
(C) Feature plots showing the expression of *ASPM*, *TOP2A*, *CCNA1*, and *CDC25B* in cycling progenitors (Cluster 5 and 6 in Figure 2A).
(D) Feature plots showing the expression of *BAX*, *BCL21*, *GADD45A*, and *XIAP* in cycling progenitors (Cluster 5 and 6 in Figure 2A).
(E) Feature plots showing the expression of *SOX2 and PAX6* in cycling progenitors (Cluster 5 and 6 in Figure 2A).
(F) Feature plots showing the expression of *FOXG1 and EMX1* in cycling progenitors (Cluster 5 and 6 in Figure 2A).
