# Supplementary material for: Divergence between neural and retinal lineage specification during human brain development by signal transduction
Source: J Adv Res. 2025 Oct 22;85:375–88. doi: 10.1016/j.jare.2025.10.034 (PMC13316595; doi:10.1016/j.jare.2025.10.034)
Supplement: Supplementary Data 4 [file mmc4.pdf]

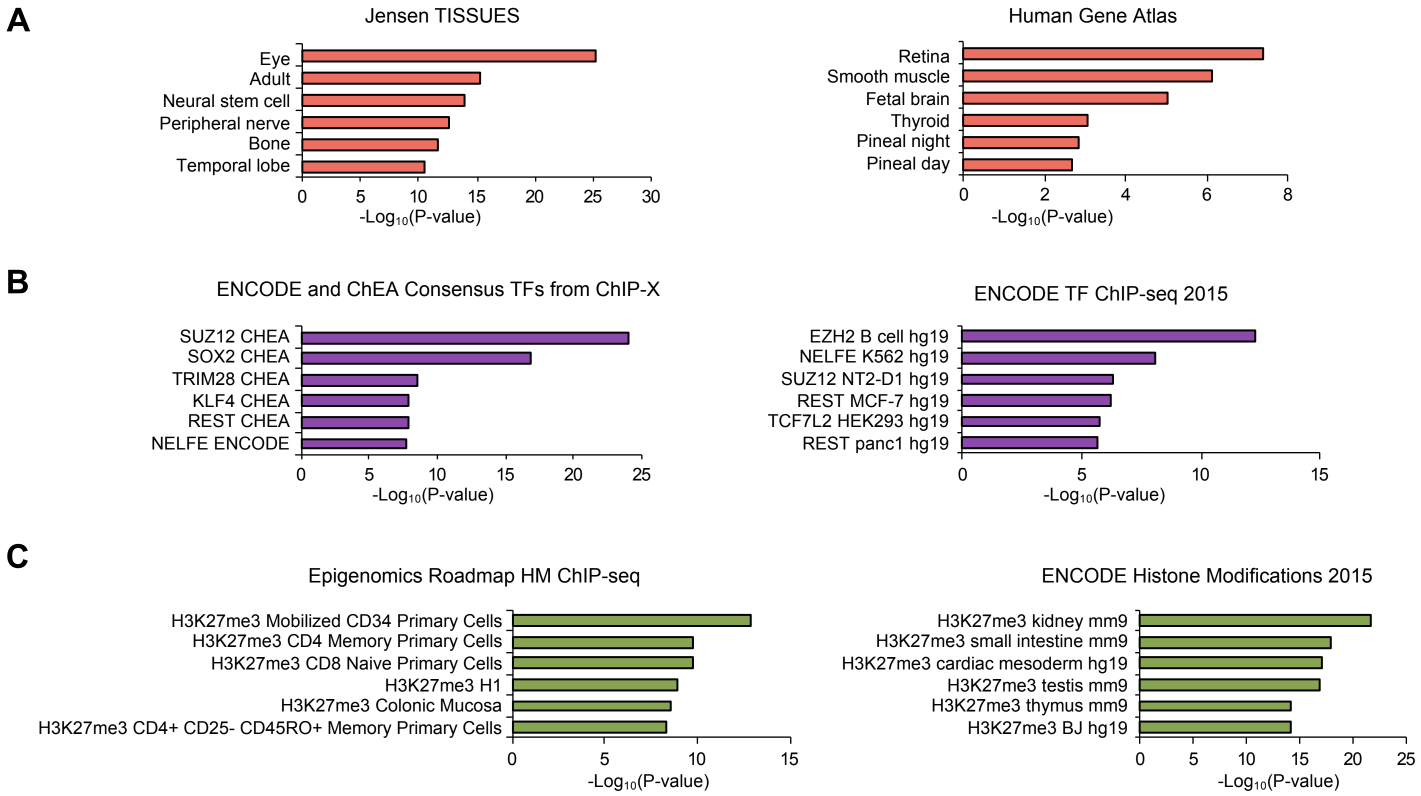

Fig. S3. Transcriptome analysis of S6K1-depleted dorsal forebrain organoids grown up to 5 weeks at single cell level.
(A) Enrichment analysis of Jensen TISSUES (left) and Human Gene Atlas (right) by EnrichR for specifically expressed genes in retinal lineage populations (Cluster 2, 3, and 4 in Figure 2A).
(B) Enrichment analysis of ENCODE and ChEA Consensus TFs from ChIP-X (left) and ENCODE TF ChIP-seq 2015 (right) by EnrichR for specifically expressed genes in retinal lineage populations (Cluster 2, 3, and 4 in Figure 2A).
(C) Enrichment analysis of Epigenomics Roadmap HM ChIP-seq (left) and ENCODE Histone Modifications 2015 (right) by EnrichR for specifically expressed genes in retinal lineage populations (Cluster 2, 3, and 4 in Figure 2A).
All graphs display top 6 categories based on p-values provided from Enrichr.
