# Supplementary material for: Divergence between neural and retinal lineage specification during human brain development by signal transduction
Source: J Adv Res. 2025 Oct 22;85:375–88. doi: 10.1016/j.jare.2025.10.034 (PMC13316595; doi:10.1016/j.jare.2025.10.034)
Supplement: Supplementary Data 5 [file mmc5.pdf]

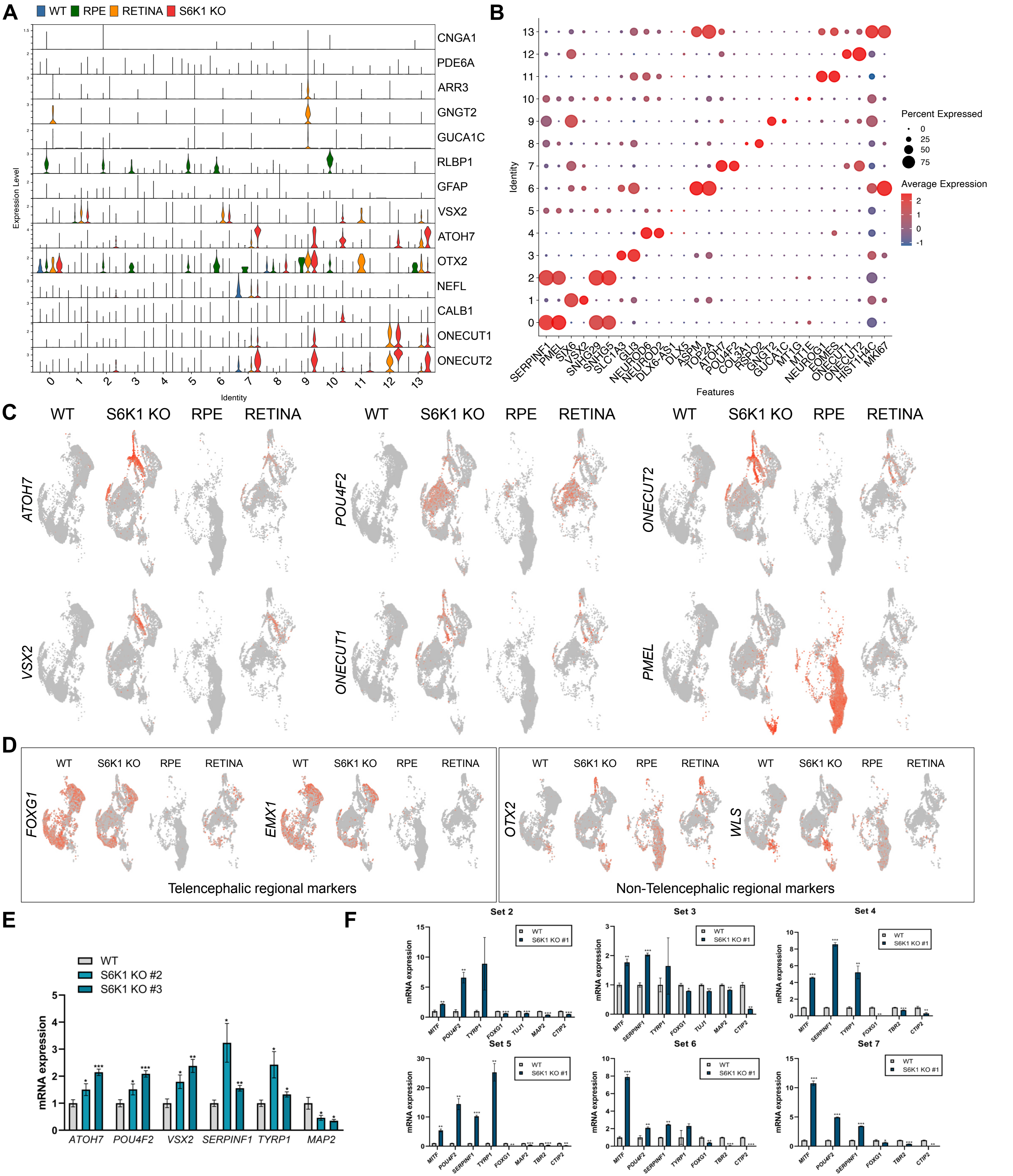

Fig. S4. Transcriptome analysis of dorsal forebrain organoids at 5 weeks (WT and S6K1 KO), human RPE, and human retina.
(A) Violin plot showing relative expression of differentially expressed genes for each cluster in single cell RNA sequencing analysis of dorsal forebrain organoids at 5 weeks (WT and S6K1 KO), human RPE, and human retina.
(B) Dot plot showing relative expression of differentially expressed genes across clusters in single cell RNA sequencing analysis of dorsal forebrain organoids at 5 weeks (WT and S6K1 KO), human RPE, and human retina.
(C) Feature plots showing the expression of retinal lineage marker genes across samples.
(D) Feature plots showing the expression of telencephalic or non-telecenphalic regional markers.
(E) The mRNA levels of retinal or neuronal marker genes were analyzed through bulk RT-qPCR of dorsal forebrain organoids derived from WT and S6K1$^{-/-}$ H7 cells (clone #2 & #3) at week 5. Mean ± SEM (n = 3 or 4)
(F) The mRNA levels of retinal or neuronal marker genes were analyzed through bulk RT-qPCR of dorsal forebrain organoids derived from WT and S6K1$^{-/-}$ H7 cells (clone #1, batch 2-7) at week 5. Mean ± SEM (n = 3 or 4)
