# Supplementary material for: Divergence between neural and retinal lineage specification during human brain development by signal transduction
Source: J Adv Res. 2025 Oct 22;85:375–88. doi: 10.1016/j.jare.2025.10.034 (PMC13316595; doi:10.1016/j.jare.2025.10.034)
Supplement: Supplementary Data 6 [file mmc6.pdf]

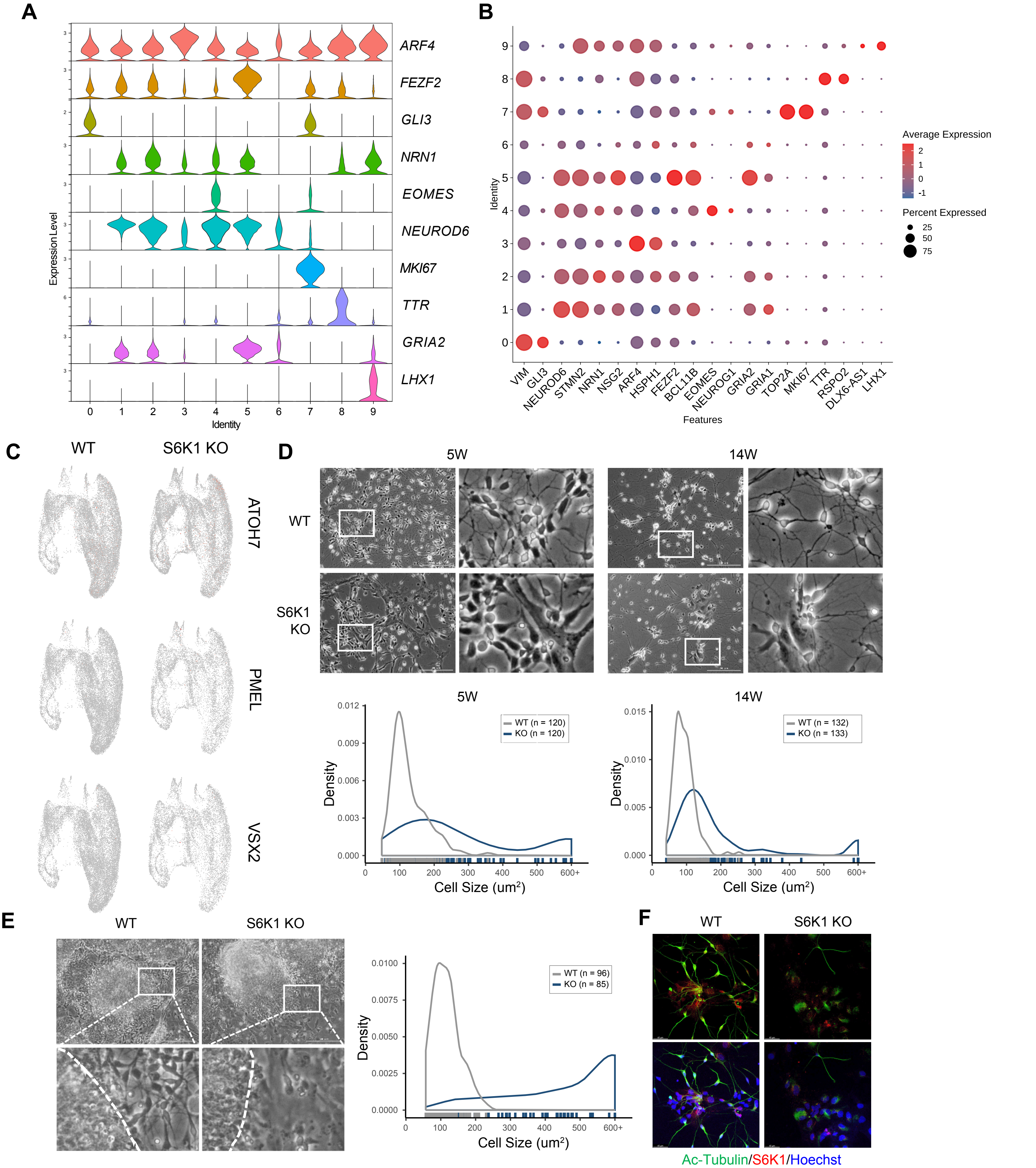

Fig. S5. Transcriptome analysis of S6K1-depleted dorsal forebrain organoids grown up to 14 weeks at single cell level.
(A) Violin plot showing relative expression of differentially expressed genes for each cluster in single cell RNA sequencing analysis at week 14.
(B) Dot plot showing relative expression of differentially expressed genes across clusters in single cell RNA sequencing analysis at week 14.
(C) Feature plots showing the expression of retinal markers ATOH6, PMEL, and VSX2.
(D) Microscopic images (upper) and distribution plot of cell size (um^2) (lower) of dissociated cells from wild-type (WT, n = 120 (5W), 132 (14W)) and S6K1 knockout (KO, n = 120 (5W), 133(14W)) brain organoid (5 or 14 weeks).
(E) Microscopic images (left) and distribution plot of cell size (um^2) (right) of monolayered cortical neurons differentiated from wild-type (WT, n = 96) and S6K1 knockout (KO, n = 85) H7 cells at day 26. Boundaries of neural rosette are presented as magnified image.
(F) Immunocytochemistry of monolayered cortical neurons differentiated from wild-type (WT) and S6K1 knockout (KO) H7 cells at day 37.
