# Supplementary material for: Divergence between neural and retinal lineage specification during human brain development by signal transduction
Source: J Adv Res. 2025 Oct 22;85:375–88. doi: 10.1016/j.jare.2025.10.034 (PMC13316595; doi:10.1016/j.jare.2025.10.034)
Supplement: Supplementary Data 7 [file mmc7.pdf]

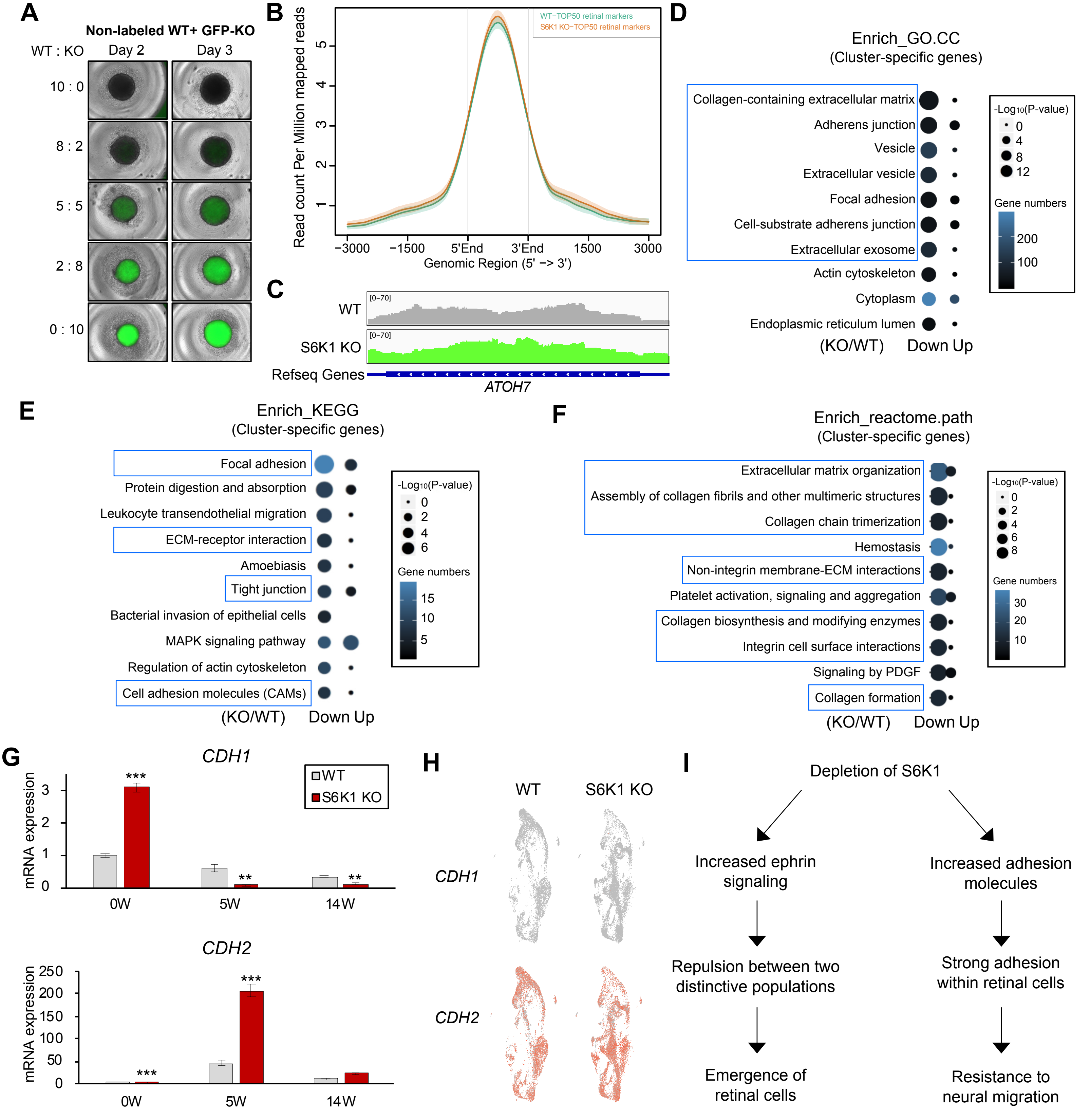

Fig. S6. S6K1 depletion dysregulates extracellular matrix generation

(A) Mixed embryonic bodies without neural induction (Day 2 or 3) using non-labeled WT and GFP labeld S6K1 KO cells with different cell ratio.

(B) Normalized read counts per million mapped reads for ATAC-seq in GFP-positive (S6K1 KO) population and GFP-negative (WT) population.

(C) IGV genome browser view of ATAC-seq signals on ATOH7 gene region.

(D, E, F) Enrichment of Gene ontology cellular components (GO.CC) (D), Enrich_KEGG (E), and Enrich_reactome.path (F) for up/down-regulated genes upon S6K1 deletion in H7 cells.

(G) The mRNA levels of *CDH1* and *CDH2* were analyzed through bulk RT-qPCR of dorsal forebrain organoids derived from WT and S6K1 KO H7 cells at week 0, 5, and 14.

(H) Feature plots showing the relative expression of *CDH1* and *CDH2* across clusters in single cell RNA sequencing analysis with dorsal forebrain organoids derived from WT and S6K1 KO H7 cells at week 5.

(I) Overview for the effect of S6K1 depletion in human brain organoids.
